# Desvenlafaxine-Triggered Acneiform Eruptions on the Hand: A Compelling Case Report

**DOI:** 10.7759/cureus.52185

**Published:** 2024-01-12

**Authors:** Anupam S Yadav, Sonali Singh, Jaismeen Randhawa, Chinaza M Akuma, Ogbonnaya Akuma, Hassan A Chaudhry

**Affiliations:** 1 Psychiatry, Ganesh Shankar Vidyarthi Memorial (GSVM) Medical College, Kanpur, IND; 2 Pediatric, King George's Medical University, Lucknow, IND; 3 Psychiatry, Sri Guru Ram Das Institute of Medical Sciences and Research, Amritsar, IND; 4 Public Health, Chamberlain University, College of Health Professions, Chicago, USA; 5 Internal Medicine, Ebonyi State University, Abakaliki Nigeria, Abakaliki, NGA; 6 Biological Sciences, Temple University, Philadelphia, USA; 7 Medicine, Medical University of Lublin, Lublin, POL; 8 Interdisciplinary Medicine, Independent Research Scholar, Philadelphia, USA

**Keywords:** snri, drug induced, skip eruptions, antidepressant, desvenlafaxine

## Abstract

A 45-year-old male developed a skin eruption after starting Desvenlafaxine for depressive symptoms associated with schizophreniform disorder. The patient developed a rash on the hand, hyperpigmentation, and itching, which resolved after discontinuing the medication. The Naranjo score suggested a probable link between desvenlafaxine and the skin reaction. Stable vital signs and normal labs supported this conclusion. The case underscores the importance of recognizing and reporting adverse drug reactions, even with generally safe medications like desvenlafaxine. Further research with larger samples is needed to explore this relationship in more depth.

## Introduction

Drug-induced skin reactions, also known as drug eruptions, can be categorized as simple/mild or complex/severe based on their potential to cause harm. Simple drug reactions typically manifest as rashes, with one common type being exanthem. These rashes tend to appear within 7 to 10 days after starting a medication. Drug exanthems are the most frequent skin reactions to medications and can present as morbilliform or maculopapular reactions on the skin [1].

Among psychotropic drugs, desvenlafaxine is a commonly prescribed SNRI (dual serotonin and norepinephrine reuptake inhibitor) [2]. While it is generally well-tolerated, adverse cutaneous reactions associated with desvenlafaxine are extremely rare, with only a limited number of cases documented in the medical literature. The reported incidence of drug-induced skin reactions linked to psychotropic drugs is low, at 0.1%. Among these reactions, antidepressants account for 29%. The likelihood of experiencing a rash or pruritus (itchiness) varies among different psychotropic drugs, with bupropion having the highest rate at 3.7% and fluoxetine, paroxetine, sertraline, and venlafaxine having the lowest rates, all less than 1% [3].

Interestingly, there have been very few reports of skin reactions associated with desvenlafaxine. Therefore, we present a case report of a middle-aged man who developed drug-induced skin eruptions due to desvenlafaxine.

## Case presentation

A 45-year-old male presented at the psychiatric outpatient department with a three-month history of anxiety, social withdrawal, suspiciousness, forgetfulness, sleep disturbances, and reduced appetite, without any identifiable precipitating factor. During the mental status examination, he exhibited reduced eye contact, slowed physical movements, brief and hesitant speech, delusions of persecution, and limited emotional expression. He was diagnosed with schizophreniform disorder as per DSM-5 criteria and initiated on a daily dose of 5 mg of olanzapine, which was gradually titrated to 10 mg per day over the next four to six weeks.

In the ensuing 1.5 months, notable amelioration of symptoms was noted. Nonetheless, the patient subsequently reported enduring fatigue, bodily discomfort of the body being too heavy and lethargic, persistent, albeit not overwhelming, anxiety, a low mood, and reduced interest in previously enjoyable activities. These emerging symptoms may be ascribed to either the negative manifestations of schizophrenia or the onset of concurrent depressive symptoms. A more thorough evaluation of the symptom progression and its nature was suggestive of a depressive origin [4].

During a follow-up visit at three months, the patient was initiated on a morning dose of 50 mg of desvenlafaxine to address the aforementioned complaints along with the ongoing treatment. Notably, he had no prior history of allergies, drug reactions, or medical illnesses. Approximately one week after initiating the medication, a rash appeared on the extensor aspect of his hand. Despite this, the patient continued to take desvenlafaxine, and within the subsequent week, he observed a worsening of the rash on his hand, accompanied by hyperpigmentation, acne-like lesions, and itching. Consequently, he discontinued the medication and noted a mild reduction in itching without further exacerbation of the rash when he returned for a review.

**Figure 1:**
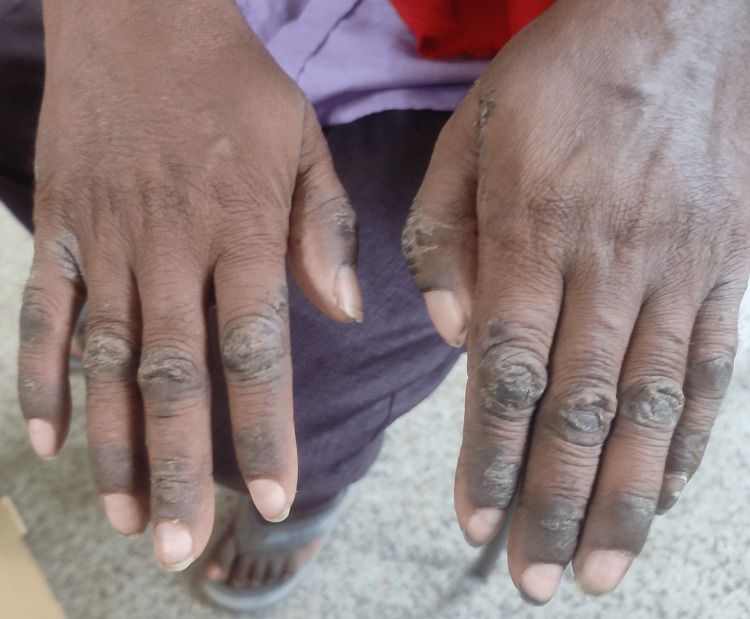
Exanthematous rash comprising papules, hyperpigmentation mostly on the extensor aspect of proximal and distal inter-phalangeal joints, intertriginous spaces and mild localized erythema.

Upon examination, an exanthematous rash comprising papules, hyperpigmentation mostly on the extensor aspect of proximal and distal inter-phalangeal joints, and mild localized erythema was observed, as illustrated in Figure 1. Notably, this condition was confined to the affected area, with no involvement of the oral or conjunctival mucosa. The patient did not exhibit any fever, joint swelling, or any other tissue involvement. Vital signs were stable, with only a slight increase in local temperature. A comprehensive systemic examination yielded results within normal parameters. Consequently, the patient was referred to the dermatology department for a professional assessment and treatment of the rash.

**Figure 2:**
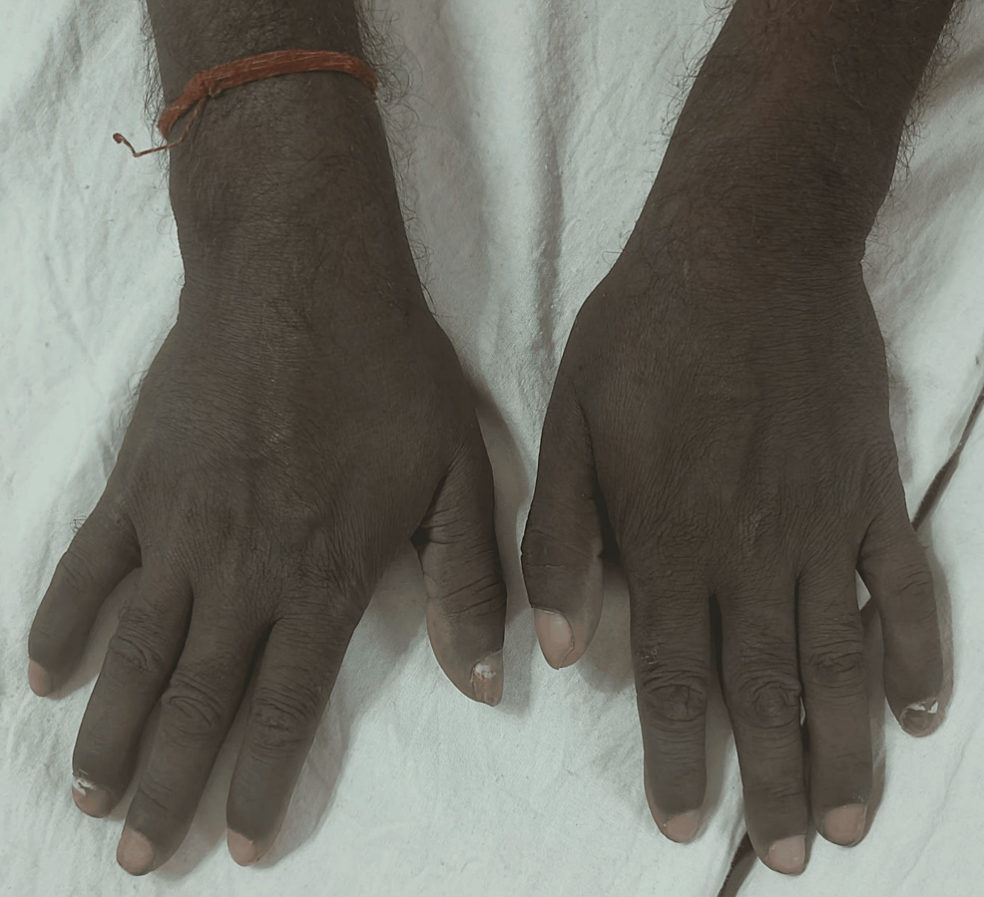
Two weeks after complete cessation of desvenlafaxine.

The diagnosis rendered by the dermatology team was drug-induced exanthem, leading to the prescription of a local steroid ointment and antihistamine medication. Laboratory investigations, including a complete blood count and liver and renal function tests, revealed values within the normal range (TLC: 10,770 cells/mm^3^, DLC: neutrophils: 72%, lymphocytes: 22%, eosinophils: 4%, monocytes: 2%, basophils: 0%, IgE: 176 IU/mL) except for an elevated erythrocyte sedimentation rate (ESR: 73 mm/h), which was monitored to rule out organ involvement and gauge severity. Importantly, there was no history of recent cosmetic product usage, local application of creams or lotions on the face, dietary changes, or any other medication that could be linked to the condition. The patient noted the rash and its associated itching subsided within two weeks following the complete cessation of desvenlafaxine (Figure 2, Table 1).

**Table 1:** Naranjo Scoring for the current case. Naranjo et al. [5].

Naranjo scoring				
Question	Yes	No	Do Not Know	Score
1. Are there previous conclusive reports on this reaction?	+1	0	0	+1
2. Did the adverse event appear after the suspected drug was administered?	+2	-1	0	+2
3. Did the adverse reaction improve when the drug was discontinued or a specific antagonist was administered?	+1	0	0	+1
4. Did the adverse reaction reappear when the drug was readministered?	+2	-1	0	0
5. Are there alternative causes (other than the drug) that could on their own have caused the reaction?	-1	+2	0	+2
6. Did the reaction reappear when a placebo was given?	-1	+1	0	0
7. Was the drug detected in the blood (or other fluids) in concentrations known to be toxic?	+1	0	0	0
8. Was the reaction more severe when the dose was increased, or less severe when the dose was decreased?	+1	0	0	0
9. Did the patient have a similar reaction to the same or similar drugs in any previous exposure?	+1	0	0	0
10. Was the adverse event confirmed by any objective evidence?	+1	0	0	0
Total				+6

Since we chose not to reintroduce Desvenlafaxine in case the rash reappeared, given the patient's Naranjo score of 6, we determined and reported the presumed drug reaction as probable. A score in the range of 5-8 indicates a probable adverse drug reaction [5]. The patient's depressive symptoms were managed with psychotherapeutic interventions in the following follow-up sessions, due to the patient's low tolerance for the SNRI and reluctance to try any other medication.

## Discussion

The emergence of the rash and associated symptoms following the initiation of desvenlafaxine establishes a temporal relationship and strongly suggests an adverse drug reaction. The Naranjo score of 6, indicating a probable relationship between the drug and the adverse event, supports this conclusion. The fact that the rash improved upon discontinuation of desvenlafaxine further reinforces the likelihood of a drug-induced reaction. It is crucial for healthcare providers to be vigilant about adverse events and conduct thorough causality assessments to ensure patient safety.

It is noteworthy that the patient's vital signs remained stable throughout the course of the adverse event, and systemic involvement was absent. Laboratory investigations, including a complete blood count and liver and renal function tests, were within normal parameters, except for an elevated ESR. This comprehensive evaluation ruled out organ involvement and helped gauge the severity of the reaction. Regular monitoring and appropriate laboratory assessments are essential to effectively managing adverse drug reactions.

Discontinuation of the offending medication, desvenlafaxine, was a crucial step in managing the adverse event. Given the likelihood of a recurrence, it was prudent not to reintroduce the drug. This decision aligns with the principle of avoiding potentially causative agents in cases of suspected drug-induced reactions. Additionally, the patient's depressive symptoms were addressed through psychotherapeutic interventions after discontinuing desvenlafaxine, highlighting the importance of considering alternative treatment strategies when adverse reactions limit the use of a particular medication.

Cutaneous drug reactions can stem from either immunological or non-immunological mechanisms, or sometimes their origin remains unknown. These reactions often result from the release of inflammatory mediators by mast cells, which can occur either directly or via IgE-specific antibodies. Additionally, the production and accumulation of toxic drug metabolites can also contribute to these reactions [6].

Serotonin plays a pivotal role in the intricate interplay between the neuroendocrine system and the skin. Platelets are the primary source of serotonin in the skin, releasing it when activated. This released serotonin interacts with membrane-bound serotonin (5-HT) and serotonin transporter receptors present in skin cells. The nature, intensity, and duration of the cutaneous serotonergic response, whether it is an intended effect or an unintended side effect, depend on this interaction [7].

Research conducted in animal studies has identified one mechanism responsible for SSRI-induced pruritis, which involves the mediation of the 5-hydroxytryptamine (HT) receptor 7 and the TRPA1 (transient receptor potential ankyrin 1) ion channel [8].

It is important to acknowledge the limitations of this case report. The Naranjo score, while a valuable tool, relies on subjective assessments and may not definitively establish causality. Furthermore, the reported reaction, along with other probable side effects, should be evaluated in a study with a large sample size and multiple sites.

## Conclusions

Desvenlafaxine is a commonly prescribed antidepressant medication, primarily attributable to its notable efficacy, well-tolerated nature, and favorable safety profile. Skin reactions induced by desvenlafaxine are exceptionally rare, with only a few case reports in the literature, despite its widespread use as an antidepressant.

Recognizing and diligently reporting drug-related side effects and adverse drug reactions, whether they are of a serious or non-serious nature, holds profound importance. Such vigilance and documentation are instrumental in facilitating a comprehensive understanding, review, and dissemination of drug-related information.
